# Should Medical Practitioners Dispense?

**Published:** 1910-06-25

**Authors:** 


### II.—THE PHARMACISTS POINT OF VIEW.

By A PHARMACEUTICAL CHEMIST.

The tendency of a modern chemist's shop to become a general store where the sale of drugs and the compounding of medicines is subsidiary to the selling of a heterogeneous collection of toilet articles and nostrums is due to two main causes—namely, the dispensing of medicines by medical practitioners and the demand for advertised quack remedies.

### The Case for the Chemist.

The attempt which is being made by representatives of the British Medical Association and the British Pharmaceutical Conference to find some practicable plan by which the dispensing of medicines could be restricted to pharmacists is, therefore, regarded with particular interest by chemists and druggists as a body, although many of them are not very hopeful that a satisfactory scheme will be evolved. Pharmacists claim that they are the only class specially educated and properly trained to dispense medicines, and yet a large proportion of them seldom have cause to put their skill into practice, for the reason that in many districts medical men do their own dispensing. Before a candidate can enter for the qualifying examination as a pharmacist he is required to produce a certified declaration that for three years he has been practically engaged in the translation and dispensing of prescriptions, and the examination is an exceedingly severe test of the candidate's knowledge of the special subjects allied to the practice of pharmacy. On the other hand, the practical knowledge of the compounding of drugs possessed by the average candidate for medical degrees is, by comparison, of a very meagre description, and the tendency with some of the examining boards is to pay less and less attention to this subject. From the standpoint of specialised training the pharmacist, therefore, has an excellent case.

### What is Prescribing?

As to the cognate subject of "prescribing" by chemists, the questions arise, "What is prescribing?" and "To what extent do chemists prescribe?" It has been urged—and there would seem to be some reasonableness in the stipulation—that if all dispensing is to be transferred to pharmacists, something must be offered as a compensation: that is to say, chemists must cease to prescribe. Of course, practices differ very considerably in different localities, and what may be repugnant to many is possibly done by a few without compunction; but if by saying that chemists prescribe it is intended to convey the impression that chemists as a body make a practice of affecting to diagnose diseases and of treating each "patient" as a special case, it is quite wrong to say that chemists prescribe. It may be done in a few cases, but the vast majority of pharmacists do not usurp the functions of the qualified medical practitioner in this way.

### Stock Remedies.

If, on the other hand, it is called prescribing to keep in stock a series of simple remedies for minor ailments, and to sell them to customers without pretending that they are anything but "stock" remedies, then chemists as a class do "prescribe." It would be extremely difficult to induce chemists to refrain from selling what their customers reasonably demand, and if the solution of the dispensing question depends on chemists ceasing to sell common remedies, the problem will probably not be solved in our time. Logically it may be improper for any druggist to sell a drug except on the order of a medical practitioner, but it would be a matter of extreme difficulty to bring the public round to this view. In selling these simple preparations the chemist is competing with the advertiser of quack nostrums and not with the qualified practitioner, for should the chemist refuse to give his customer what he asked for the customer would in all probability buy instead one of the largely advertised quack remedies. The whole question bristles with difficulties, and if the British Medical Association and the British Pharmaceutical Conference can suggest some reasonable way of overcoming these difficulties, pharmacists will be grateful to them.

